# Antioxidant effects of *Dendropanax morbifera* Léveille extract in the hippocampus of mercury-exposed rats

**DOI:** 10.1186/s12906-015-0786-1

**Published:** 2015-07-23

**Authors:** Woosuk Kim, Dae Won Kim, Dae Young Yoo, Hyo Young Jung, Jong Whi Kim, Dong-Woo Kim, Jung Hoon Choi, Seung Myung Moon, Yeo Sung Yoon, In Koo Hwang

**Affiliations:** Department of Anatomy and Cell Biology, College of Veterinary Medicine, and Research Institute for Veterinary Science, Seoul National University, Seoul, 151-742 South Korea; Department of Biochemistry and Molecular Biology, Research Institute of Oral Sciences, College of Dentistry, Kangneung-Wonju National University, Gangneung, 210-702 South Korea; Central Research Center, Egreen Co. Ltd, Seongnam, 463-862 South Korea; Department of Anatomy, College of Veterinary Medicine, Kangwon National University, Chuncheon, 200-701 South Korea; Department of Neurosurgery, Dongtan Sacred Heart Hospital, College of Medicine, Hallym University, Hwaseong, 445-170 South Korea

**Keywords:** *Dendropanax morbifera* extract, Mercury, Hippocampus, Reactive oxygen species, Protein modification

## Abstract

**Background:**

*Dendropanax morbifera* Léveille has been employed for the treatment of infectious diseases using folk medicine. In this study, we evaluated the antioxidant effects of a leaf extract of *Dendropanax morbifera* Léveille in the hippocampus of mercury-exposed rats.

**Methods:**

Seven-week-old Sprague–Dawley rats received a daily intraperitoneal injection of 5 μg/kg dimethylmercury and/or oral *Dendropanax morbifera* Léveille leaf extract (100 mg/kg) for 4 weeks. Animals were sacrificed 2 h after the last dimethylmercury and/or leaf extract treatment. Mercury levels were measured in homogenates of hippocampal tissue, a brain region that is vulnerable to mercury toxicity. In addition, we measured reactive oxygen species production, lipid peroxidation levels, and antioxidant levels in these hippocampal homogenates.

**Results:**

Treatment with *Dendropanax morbifera* Léveille leaf extract significantly reduced mercury levels in hippocampal homogenates and attenuated the dimethylmercury-induced increase in the production of reactive oxygen species and formation of malondialdehyde. In addition, this leaf extract treatment significantly reversed the dimethylmercury-induced reduction in the hippocampal activities of Cu, Zn-superoxide dismutase, catalase, glutathione peroxidase, and glutathione-*S*-transferase.

**Conclusion:**

These results suggest that a leaf extract of *Dendropanax morbifera* Léveille had strong antioxidant effects in the hippocampus of mercury-exposed rats.

## Background

Recently, several attempts have been made to develop approaches that facilitate the removal of heavy metals from the body, because these metals have been found to accumulate in the body over time [1–4]. The accumulation of heavy metals such as mercury (Hg), lead, and cadmium can cause dangerous conditions, including neurological dysfunction and metabolic disorders [5]. Organic Hg can accumulate due to exposure to dimethylmercury (MeHg) and this is the most common cause of intoxication in humans [6]. The main cause of Hg exposure in humans and animals is the consumption of fish containing MeHg [7, 8]. Although MeHg has low lipid solubility, the ingested MeHg easily permeates the blood–brain barrier and accumulates in the hippocampus [9], a brain region that is vulnerable to acute MeHg exposure [10]. In a recent study, MeHg was found to induce the accumulation of amyloid-β in the brain and facilitate the progression of Alzheimer’s disease [11].

Strong chelating agents such as ethylenediaminetetraacetic acid (EDTA) can efficiently remove heavy metals from contaminated soils. However, the toxicity of EDTA limits its application in animals or humans.

Recently, there have been many attempts to exploit the antioxidant potential of compounds identified in plants [12–16]. We previously demonstrated that a stem extract of *Dendropanax morbifera* facilitated the excretion of cadmium and increased antioxidant levels in the hippocampus [17]. In addition, *Dendropanax morbifera* extract increased the activities of antioxidant enzymes and showed protective effects against diabetes, cancer, atherosclerosis, and kidney toxicity [18–21]. However, there are no reports on the effects of *Dendropanax morbifera* extracts on the hippocampal antioxidant status after MeHg intoxication. Therefore, this study examined the effects of an extract of *Dendropanax morbifera* leaves (DML) on oxidative stress and the status of antioxidant enzymes in the hippocampus of rats exposed to MeHg.

## Methods

### Experimental animals

Male Sprague–Dawley rats were purchased from Orient Bio Inc. (Seongnam, South Korea). Rats were housed in a conventional animal facility at 23 °C with 60 % humidity, a 12 h/12 h light/dark cycle, and free access to food and tap water. The handling and care of the animals conformed to the guidelines established in order to comply with current international laws and policies (NIH Guide for the Care and Use of Laboratory Animals, NIH Publication No. 85–23, 1985, revised 1996). Ethical and experimental protocol approvals were obtained from the Institutional Animal Care and Use Committee (IACUC) of Seoul National University (Approval number: SNU-130522-1). All of the experiments were conducted with an effort to minimize the number of animals used and the suffering caused by the procedures employed.

### Preparation of DML

Fresh *Dendropanax morbifera* Léveille was purchased from a local market on Jeju Island in Korea. The plant was authenticated by two practitioners of traditional Asian medicine, and a voucher specimen was deposited with Egreen Co. Ltd. (deposition number: 2013–002). Leaves from the plant samples (100 g) were chopped, blended, soaked in 2 L 80 % ethanol, and then refluxed three times at 20 °C for 2 h. The insoluble materials were removed by centrifugation at 10,000 × *g* for 30 min, and the resulting supernatant was concentrated and freeze-dried to yield a powder. Before each experiment, the dried extract was dissolved in distilled and deionized water.

### Administration of MeHg and DML

MeHg was purchased from Sigma-Aldrich (St. Louis, MO, USA). Animals were divided into 4 treatment groups (*n* = 21 in each group): 1) a control group received oral distilled water and intraperitoneal injections of physiological saline, 2) a DML group was treated with 100 mg/kg oral DML and intraperitoneal injections of physiological saline, 3) a MeHg group was administered with oral distilled water and intraperitoneal injections of 5 μg/kg MeHg, and 4) a DML-MeHg group received 100 mg/kg oral DML and intraperitoneal injections of MeHg. MeHg was administered intraperitoneally and DML was administered orally to 7-week-old rats once a day for 4 weeks.

### Hg levels in hippocampal homogenates

Rats in the control, DML, MeHg, and MeHg-DML groups (*n* = 7 from each group) were anesthetized with 1 g/kg urethane (Sigma-Aldrich) and the hippocampi were dissected out to measure the accumulation of Hg in the hippocampal homogenates. Hippocampi were weighed in glass vessels, and tissues were digested by adding 3-8 mL of HNO_3_ for 3 h, after which 2 mL of H_2_O_2_ was added and the samples were heated for 1 h. Digested hippocampal samples were transferred to polypropylene flasks for Hg determination, which was performed using inductively coupled plasma mass spectrometry (ICP-MS; PerkinElmer Sciex, Thornhill, Canada).

### Measurement of reactive oxygen species (ROS) production and lipid peroxidation in the hippocampus

The effects of DML and MeHg on ROS production and lipid peroxidation were determined in hippocampal homogenates from rats in the control, DML, MeHg, and MeHg-DML groups (*n* = 7 from each group) using the fluorescent probe, 2ʹ,7ʹ-dichlorofluorescin diacetate (DCFH-DA) [22], and by measuring malondialdehyde (MDA) formation, respectively. Intracellular ROS oxidize DCFH-DA to dichlorofluorescein (DCF), an intensely fluorescent chemical. The rats were deeply anesthetized with urethane and euthanized by decapitation after treatment for 4 weeks. Bilateral hippocampi were dissected out and the left and right parts were used to measure ROS production and lipid peroxidation, respectively. Hippocampal mitochondria were obtained as described previously [23]. Mitochondrial protein was quantified by the Bradford method [24] using bovine serum albumin (BSA) as the standard. The isolated mitochondria (0.5 mg protein/mL) were incubated with 10 μM DCFH-DA at 37 °C for 60 min, and the fluorescence intensity of DCF was measured at an excitation wavelength of 488 nm and emission wavelength of 527 nm in a microplate reader (SpectraMax M5, Molecular Devices LLC, Sunnyvale, CA).

To measure MDA production, the hippocampal tissues were homogenized in 20 mM phosphate buffered saline (pH 7.4) containing 5 mM butylated hydroxytoluene. After centrifugation of the homogenates at 3000 × *g* for 10 min at 4 °C, the supernatants were collected. For each reaction, 10 μL of probucol and 640 μL of diluted R1 reagent (1:3 of methanol:*N*-methyl-2-phenylindole) were added and mixed with 150 μL of 12 N HCl. Each reaction was incubated at 45 °C for 60 min and then centrifuged at 10,000 × *g* for 10 min. The supernatant was collected and MDA formation was determined by measuring the absorbance at 586 nm. MDA data were normalized to the protein concentration of each sample.

### Measurement of antioxidant enzyme activity in hippocampal homogenates

To elucidate the effects of DML and MeHg on Cu, Zn-superoxide dismutase 1 (SOD1), catalase (CAT), glutathione peroxidase (GPx), and glutathione-related enzymes such as glutathione-*S*-transferase (GST), and glutathione reductase (GR), the activities of these enzymes were measured in rats from the control, DML, MeHg, and DML-MeHg groups (*n* = 7 from each group). Animals were deeply anesthetized with urethane and euthanized by decapitation after treatment for 4 weeks. Bilateral hippocampi were dissected out and the left part was used to measure SOD1, CAT, and GPx activities, while the right part was used to measure glutathione-related enzyme activities. Left and right hippocampal tissues were homogenized in 10 mM Tris buffer containing 1 mM EDTA or 1 mM phenylmethanesulfonylfluoride, respectively. The homogenates were centrifuged at 600 × *g* for 10 min, and then centrifuged at 13,000 × *g* for 20 min at 4 °C.

SOD1 activity was measured by monitoring its capacity to inhibit the reduction of ferricytochrome *c* by xanthine/xanthine oxidase, as described by McCord and Fridovich [25]. Protein samples were electrophoresed in 10 % native polyacrylamide gels prior to SOD1 activity visualization, as described by Beauchamp and Fridovich [26]. Briefly, the gel was soaked in 2.45 mM nitroblue tetrazolium solution for 15 min, followed by 30 min in 28 mM *N*,*N*,*N′′*,*N′′*-tetramethylethylene diamine and 28 μM riboflavin in 0.36 mM potassium phosphate buffer (pH 7.8). The gel was then exposed to a fluorescent light source until the bands showed maximum resolution.

CAT activity was assayed at 25 °C by determining the rate of H_2_O_2_ degradation in 10 mM potassium phosphate buffer (pH 7.0), according to the method described by Aebi [27]. An extinction coefficient of 43.6 mM/cm was used for the calculations. One unit was defined as consuming 1 pmol of H_2_O_2_ per min and the specific activity was reported as units/mg protein.

GPx activity was assayed by measuring nicotinamide adenine dinucleotide phosphate (NADPH) oxidation using *t*-butyl-hydroperoxide as a substrate, as described by Maral et al. [28]. Briefly, the reaction was carried out at 25 °C in 600 μL of a solution containing 100 mM potassium phosphate buffer (pH 7.7), 1 mM EDTA, 0.4 mM sodium azide, 2 mM glutathione, 0.1 mM NADPH, 0.62 U of glutathione reductase, and 50 μL of homogenate.

Total sulfhydryl (TSH) content was determined using the 5,5’-dithiobis (2-nitrobenzoic acid) method (Sigma) reported by Riddles et al. [29].

GST activity was determined spectrophotometrically using 1-chloro-2,4-dinitrobenzene as a substrate [30]. GR, which has been shown to utilize NADPH to convert oxidized glutathione (GSSG) to the reduced form (GSH), was assayed using the method reported by Horn and Burns [31].

### Statistical analysis

All data are expressed as mean ± standard error of the mean (SEM). Differences between the means were statistically analyzed using two-way analysis of variance (ANOVA) with repeated measures and Bonferroni’s post hoc test.

## Results

### Effects of MeHg and DML on hippocampal Hg levels

**Fig. 1:**
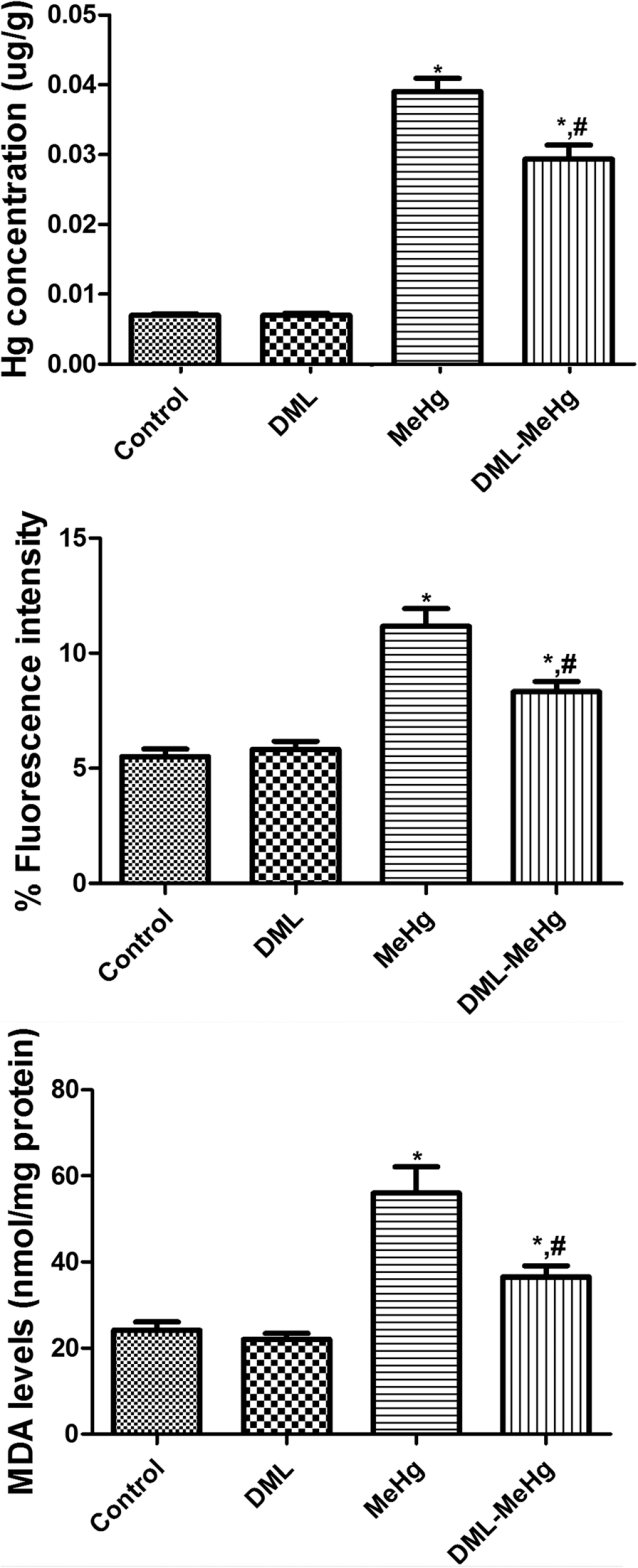
Serum mercury (Hg) levels, hippocampal production of reactive oxygen species as determined by 2′,7’-dichlorofluorescein (DCF) levels, and malondialdehyde (MDA) levels in rats. ^*^ Indicates a significant difference between the control and dimethylmercury (MeHg) groups or between the *Dendropanax morbifera* leaf extract (DML) and DML-MeHg groups (*P* < 0.05); ^*#*^ indicates a significant difference between the control and DML groups or between the MeHg and DML-MeHg groups (*p* < 0.05; *n* = 7 per group). Serum Hg, hippocampal DCF, and MDA levels are significantly increased in the MeHg group and are reduced by DML treatment. The data represent means ± standard error of the mean (SEM)

Similar hippocampal Hg concentrations were observed in the DML and control groups. The Hg concentration in the MeHg group was significantly increased by 5.57-fold, as compared with the control group. In the DML-MeHg group, the Hg concentration was significantly decreased to 75.4 % of that observed in the MeHg group, although it remained significantly elevated by 4.20-fold, as compared with the control group (Fig. 1).

### Effects of MeHg and DML on hippocampal ROS formation and lipid peroxidation

Similar DCF fluorescence intensity and MDA levels were detected in the DML and control groups. In the MeHg group, DCF fluorescence intensity and MDA levels were significantly increased to 202.5 % and 231.3 % of the control values, respectively. In the DML-MeHg group, the DCF fluorescence intensity and MDA levels were significantly decreased, as compared with those in the MeHg group (Fig. 1).

### Effects of MeHg and DML on hippocampal SOD1, CAT, and GPx activities

**Fig. 2:**
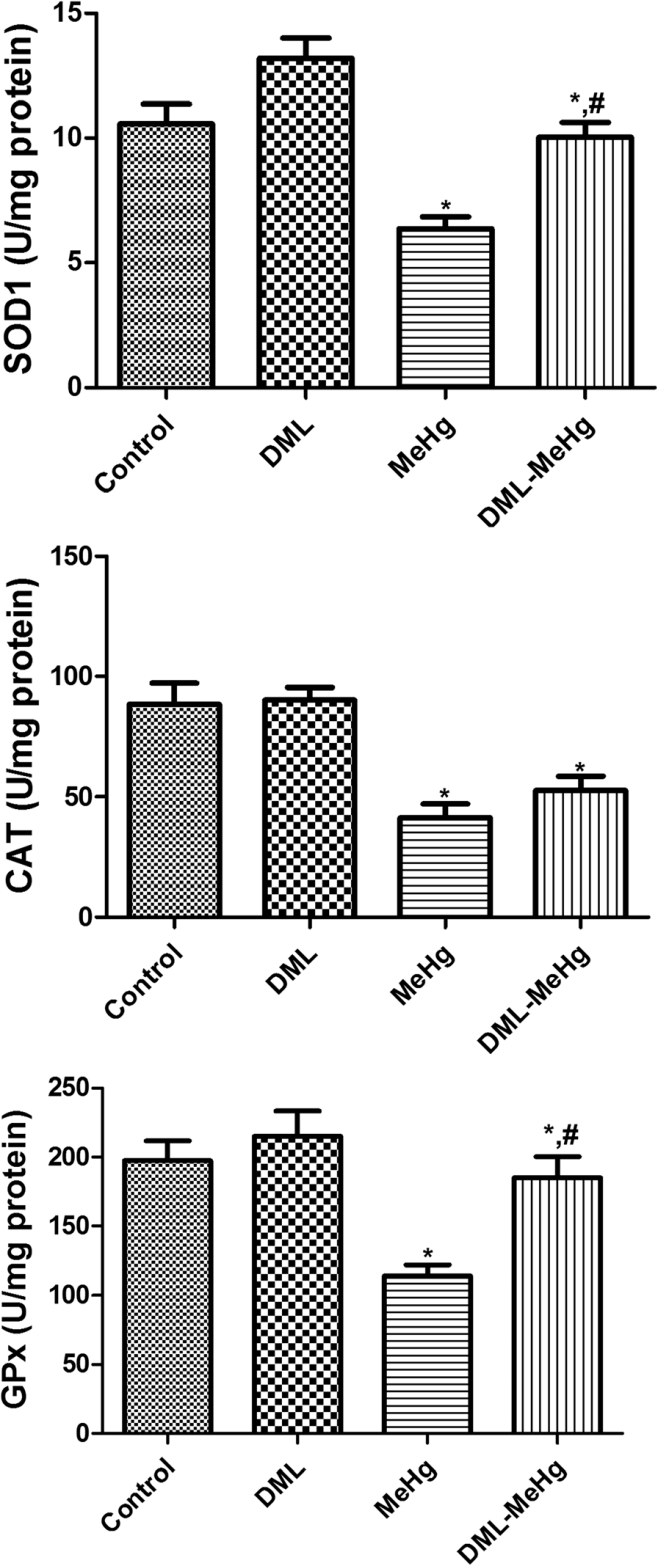
Rat hippocampal activities of Cu, Zn-superoxide dismutase 1 (SOD1), catalase (CAT), and glutathione peroxidase (GPx). ^*^ Indicates a significant difference between the control and dimethylmercury (MeHg) groups or between the *Dendropanax morbifera* leaf extract (DML) and DML-MeHg groups (*p* < 0.05); ^*#*^ indicates a significant difference between the control and DML or between the MeHg and DML-MeHg groups (*p* < 0.05; *n* = 7 per group). The activities of SOD1, CAT, and GPx are significantly decreased in the MeHg group, but DML administration significantly attenuates these effects. The data represent means ± standard error of the mean (SEM)

In the control group, the mean SOD1, CAT, and GPx activities were 10.57 U/mg protein, 88.51 U/mg protein, and 197.37 U/mg protein, respectively. In the DML group, SOD1 and GPx activities were higher than those in the control group, but these differences were not statistically significant. In contrast, CAT activity in the DML group was similar to that observed in the control group. In the MeHg group, SOD1, CAT, and GPx activities were significantly decreased to 60.3 %, 46.7 %, and 57.7 % of the control values, respectively. In the DML-MeHg group, SOD1 and GPx activities were significantly increased to 94.9 % and 93.8 % of the control values, respectively. However, CAT activity only showed a slight increase, compared with the control group (Fig. 2), and this change was not statistically significant.

### Effects of MeHg and DML on hippocampal level of TSH and activities of GST and GR

**Fig. 3:**
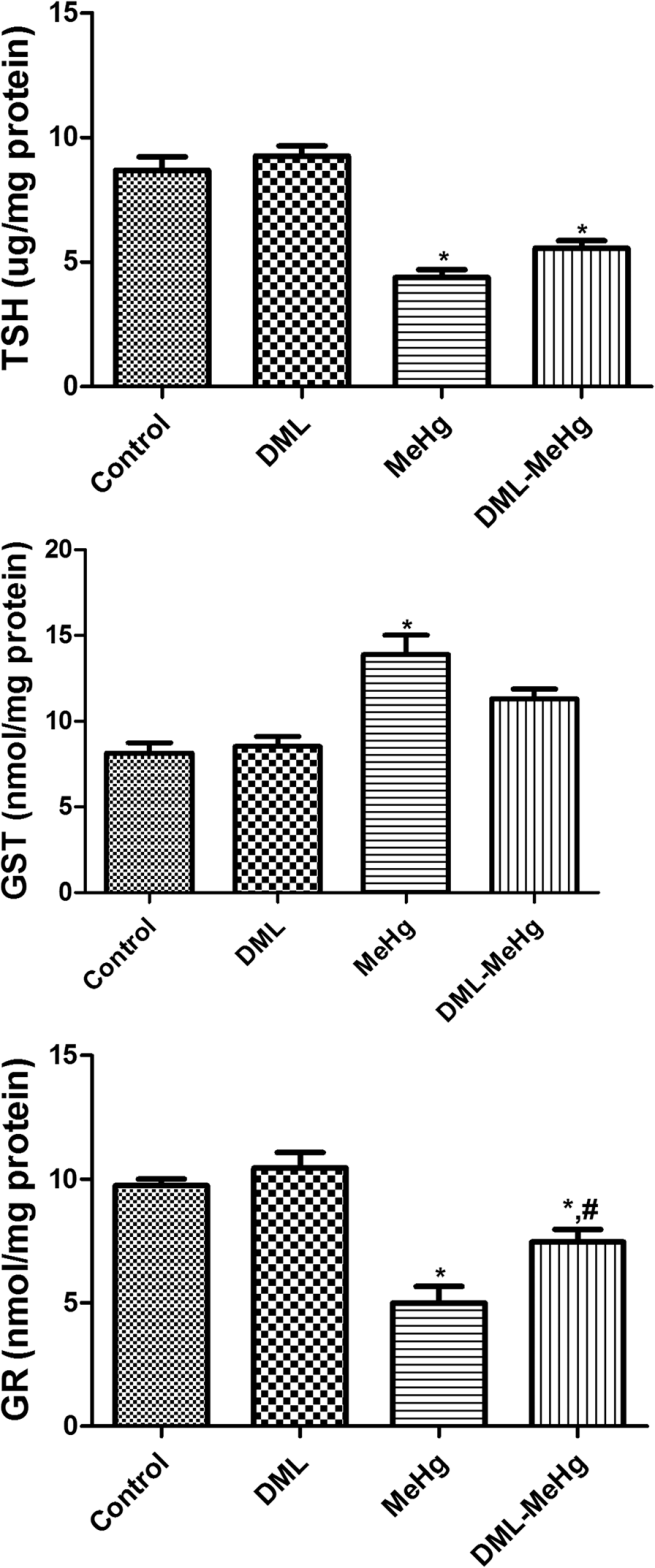
Levels of total sulfhydryl (TSH), glutathione-S-transferase (GST), and glutathione reductase (GR) in the hippocampi of control, DML, MeHg, and DML-MeHg groups following 4 weeks of DML and/or MeHg treatment. ^*^ Indicates a significant difference between the control and MeHg groups or DML and DML-MeHg groups (*p* < 0.05); ^*#*^ indicates a significant difference between the control and DML or between MeHg and DML-MeHg groups (*p* < 0.05; *n* = 7 per group). TSH levels and GR activity are significantly decreased in the MeHg group, while GST activity is significantly increased. The administration of DML reduces the changes of these parameters. The data represent means ± standard error of the mean (SEM)

The control and DML groups showed similar levels of TSH and activities of GST and GR. In the MeHg group, the TSH level and activities of GST and GR were significantly changed, as compared with the control group. The TSH level and GR activity in hippocampal homogenates were significantly decreased to 50.3 % and 51.4 % of the control values, respectively. In contrast, GST activity was significantly increased to 170.3 % of the value observed in the control group. In the DML-MeHg group, the TSH level was slightly increased compared with that in the MeHg group, while GST activity was decreased. However, these changes were not statistically significant. GR activity in the DML-MeHg group was significantly increased to 148.8 % of the value in the MeHg group (Fig. 3).

## Discussion

MeHg is a highly neurotoxic organometallic cation that poses a great risk to human health. Several lines of evidence show that its main neurotoxic mechanism involves induction of oxidative stress [32–34]. The hippocampus is particularly vulnerable to MeHg and shows detrimental changes in response to MeHg exposure [35, 36]. It has been reported that subchronic (20 days) exposure to a low concentration of Hg (1-2 mg/kg) had no direct toxic effects on the reproductive system of rats. In contrast, subcutaneous treatment of rats with 0.6 μg/g MeHg on postnatal day 7 caused spatial memory deficits on postnatal day 21, by reducing hippocampal neurogenesis [37]. In the present study, we treated rats with 5 μg/kg MeHg daily for 4 weeks and observed the effects of DML against MeHg-induced oxidative stress in the hippocampus. DML significantly reduced the MeHg-induced accumulation of Hg in hippocampal homogenates.

Next, we measured ROS production in hippocampal homogenates, because some studies demonstrated that the main mechanism of Hg toxicity in biological systems was related to the production of ROS [5, 32, 33]. In the present study, we observed that administration of MeHg significantly increased ROS formation in the hippocampus. This result was consistent with previous studies showing that MeHg significantly increased ROS production in brain synaptosomes [38] and mitochondria [39, 40]. DML administration significantly attenuated the MeHg-induced ROS production in hippocampal homogenates. This may be associated with the antioxidant properties of DML. In a previous study, we demonstrated that DML strongly reduced cadmium-induced ROS production in the hippocampus [17]. In addition, a study reported that a methanol extract of the debarked stem of *Dendropanax morbifera* had strong antioxidant activities based on its 1,1-diphenyl-2-picrylhydrazyl (DPPH) scavenging activity and ferric-reducing ability, as compared with a control material (butylated hydroxytoluene) [20]. In addition, this methanol extract of *Dendropanax morbifera* debarked stem contained abundant phenolic compounds, and the total flavonoid content was higher than that observed in extracts of the branches, bark, or yellow leaves of *Dendropanax morbifera* [20].

In the present study, we also measured the levels of antioxidant and glutathione-related enzyme activities because MeHg-induced oxidative damage reportedly decreased the levels of endogenous non-enzymatic antioxidants and inhibited antioxidant enzymes [41–46]. Conversely, depletion of glutathione facilitates MeHg accumulation and enhances MeHg-induced oxidative stress [47]. In the present study, MeHg exposure significantly decreased the activities of SOD1, CAT, and GPx in hippocampal homogenates. The reduction of antioxidant enzyme activity was most marked for CAT. The administration of DML reversed the MeHg-induced depletion of SOD1 and GPx activity to nearly the same levels as those observed in the control group. However, we did not observe any significant recovery of CAT activity. MeHg exposure significantly decreased the rat hippocampal TSH level and GR activity, while GST activity was significantly lower than that of the control group. This result was consistent with previous studies indicating that sulfhydryl groups represented the main target of MeHg in biological systems [34] and that MeHg decreased glutathione levels in the cerebellum [48]. This result was also consistent with a previous study showing that the CHCl_3_ fraction of the *Dendropanax morbifera* methanol extract exerted protective effects through its antioxidant activity, protection of mitochondria, and anti-apoptotic actions [49]. The administration of DML attenuated the changes in TSH levels and GR and GST activities in MeHg-exposed rats. This ameliorative effect of DML may reflect the increased GPx and GST activities, facilitating detoxification of the H_2_O_2_ produced by MeHg [50].

## Conclusions

In conclusion, DML significantly reduced MeHg-induced oxidative stress in the rat hippocampus by directly scavenging free radicals or by increasing the activities of antioxidant enzymes such as SOD1 and GPx.
